# Psychiatric Risk Factors for Postpartum Depression: A Systematic Review

**DOI:** 10.3390/bs15020173

**Published:** 2025-02-07

**Authors:** Renata Tambelli, Sara Tosto, Francesca Favieri

**Affiliations:** Department of Dynamic and Clinical Psychology and Health Studies, Via degli Apuli 1, 00185 Rome, Italy; tosto.1903620@studenti.uniroma1.it

**Keywords:** postpartum depression, risk factors, systematic review, psychiatric risk factors

## Abstract

The perinatal period, due to the many physical, psychological, and social changes in future mothers, may represent a critical phase with an increased risk for mental health. Postpartum depression (PPD) is one of the main syndromes that affect around 17 percent of women after pregnancy and in the first months of motherhood. This systematic review, following PRISMA guidelines, aimed to identify the main pre-partum psychiatric risk factors that may influence the occurrence and diagnosis of PPD with a focus on the antenatal and clinical history of depression, bipolar disorders, obsessive–compulsive disorder, and psychosis. From the search in main scientific databases (Web of Science, Pubmed, Psychinfo, and Scopus), 37 articles were included for the critical evaluation. The studies showed that antenatal depression and depressive episodes during pregnancy represent higher risk factors for PPD. Also, a clinical history of major depression, especially if associated with other risk factors (such as poor demographic or social conditions) increases the risk for PPD. From the systematic analysis emerged a paucity of studies considering the other psychiatric syndromes that should be overcome. PPD represents a multisystemic syndrome involving all the aspects of a mother’s life as well as affecting children’s development; for this reason, exploring the role of mental health risk factors for PPD onset, progression, and prognosis is relevant, from a clinical point of view, to find the best way to promote the mother’s psychological well-being from the antenatal period.

## 1. Introduction

Winnicott’s primary maternal preoccupation and Stern’s motherhood constellations (Bernstein & Winnicott, 1958; Stern, 2004) focused on the mental framework of the mothers who, from pregnancy to the first months of the newborn, are involved in redefining their values, interests, and priorities, developing new emotional skills to meet the needs of the child. The exploration of the perinatal period, which involves the birth of both a new human being and the sense of responsibility for their survival and well-being, drives clinicians and researchers to focus on the risks for mood alteration of mothers (Biaggi & Pariante, 2020). A certain degree of depressive symptomatology during the perinatal period is quite common in mothers, due to the inevitable restructuring that is experienced in multiple life dimensions and that is perceived differently according to an individual’s characteristics (Van Niel & Payne, 2020).

Today, the term “perinatal depression” is used to refer to the onset of depression during pregnancy or the postpartum period. Maternal depression in the postpartum period (from the 4th week to the first year of the child’s life) is recognized as postpartum depression (PPD) (APA, 2013). In the last version of the DSM-5, PPD is not recognized as a separate diagnosis but as a subtype of major depressive disorder (APA, 2013). In fact, clinically PPD is characterized by similar symptoms of major depressive disorder (e.g., sadness, marked reduction in interest in activities, agitation or psychomotor retardation, lack of energy, feelings of worthlessness and excessive guilt, and thoughts of death). Other symptoms specifically involving the bond with the child (e.g., worries about the child’s well-being, lack of attachment or interest in the child, thoughts of harming oneself or the child, active anger, and resentment towards the child) can be experienced (Van Niel & Payne, 2020). According to this large constellation of symptoms that can characterize PPD, it is characterized by highly variable conditions (Kroska & Stowe, 2020), and for this reason, it is essential to differentiate it from other forms of perinatal mood alterations, such as maternity blues and postpartum psychosis (Hamilton, 1967; Beck & Driscoll, 2006). Maternity blues has been defined as a transient and mild form of depression lasting up to two weeks, highly prevalent in new mothers: its prevalence is estimated to be around 80% (Van Niel & Payne, 2020). Its manifestation can be attributed to the physical and hormonal changes, following childbirth, such as decreased levels of thyroid hormones, estrogen, and progesterone (Tosto et al., 2023). Postpartum psychosis is a more severe but less common condition characterized by the presence of polymorphic delusions, often involving the infant, altered states of consciousness, mood disturbances, generalized anxiety, and ecstatic states. It typically has a sudden onset within the first two to three weeks postpartum and can persist for several days or weeks, but may last up to 6 months, usually with a favorable prognosis (Agrawal et al., 2022; Perry et al., 2021a).

The prevalence of PPD varies across countries, and several factors can influence its occurrence. A meta-analysis of Wang et al. (2021), including 565 studies from 80 different countries, estimated the global prevalence of postpartum depression to be approximately 17.22%. The study revealed significant differences between geographic regions, influenced by the level of development and income of the countries. The authors suggest that variation in prevalence may be influenced by cultural variations, different outcome assessment modalities, heterogeneity in the perspectives on mental disorders, the stigma surrounding mental health, especially in the context of parenting, socioeconomic class, poverty levels, limited access to public services, malnutrition, high levels of stress, and biological factors. It is clear, therefore, that beyond the diagnosis of postpartum depression, multiple demographic, social, relational, and psychological factors interact in its expression and diffusion (Wang et al., 2021; Tambelli et al., 2019). Huang et al. (2023), further exploring possible factors influencing risks and prevalence of PPD, did not report differences between women at their first pregnancy and women with previous childbirth and maternity experiences, despite in this last case where there was an increased risk for health problems and complications associated with pregnancy and childbirth due to the older age (Balasch & Gratacós, 2011; Abdollahi et al., 2014).

A higher risk of PPD seems to be associated with alcohol consumption and smoking (Filha et al., 2016). Domestic violence increases the risk of PPD (Khalifa et al., 2016), especially in cases of severe emotional violence or a combination of different forms of partner violence. Stressful life events occurring during pregnancy, after childbirth, or within the last 12 months (A. F. Bell et al., 2016), as well as high levels of perceived stress (Underwood et al., 2017), have also been found to be risk factors for the postnatal period. Several studies have identified specific psychodynamic factors underlying perinatal depression, such as poor emotional relationships with their mothers (Valverde et al., 2023), significantly more negative perceptions of the amount of care received from their parents, anxious attachment, and immature defensive style (Erickson et al., 2019; McMahon et al., 2015). The social context affects and moderates the risks for PPD. Women who report a lack of practical and emotional support, high perceived isolation, and problems within the couple during pregnancy and after childbirth are at higher risk of developing postpartum depressive symptoms (Alfayumi-Zeadna et al., 2015; de Castro et al., 2015; Du Preez et al., 2016). A recent meta-analysis (Liu et al., 2022) focused on exploring risk factors for PPD included 127 studies, highlighting six significant risk factors that increase this risk: (i) gestational diabetes, (ii) a history of depression, (iii) depression during pregnancy, (iv) giving birth to a male child, (v) receiving epidural anesthesia during childbirth, and finally, (vi) a tendency to suffer from depression during pregnancy.

Overall, this evidence suggests a multifactorial hypothesis for PPD etiopathogenesis, which should also consider psychological factors (de Castro et al., 2015).

Considering the previous premises and with the aim of further exploring the risk factors for PPD, the objective of this work is to identify and synthesize, through a systematic process, the main pre-partum psychiatric risk factors that may influence the occurrence and diagnosis of postpartum depression. In particular, the study hypothesizes the presence in the clinical history of patients with PPD of mood disorders (major depressive disorder, bipolar disorder), obsessive–compulsive disorders, and psychosis, which may represent risk factors for the onset of a depressive clinical frame after childbirth. Further, possible co-occurrent risk factors reported by the study were clarified.

## 2. Materials and Methods

### 2.1. Search Strategies

This systematic review was carried out in accordance with the PRISMA guidelines (Page et al., 2021). A systematic search in the main databases (Web of Science, Pubmed, Psychinfo, and Scopus) was conducted with a last update on 15 July 2024.

The following MeSh terms and words were combined to structure the systematic search: ‘postpartum depression OR perinatal depression OR postnatal depression OR PPD OR PND’ AND ‘bipolar disorder OR depression OR major depressive disorder OR schizophrenia OR psychotic symptom* OR obsessive–compulsive disorder OR DOC’.

**Figure 1:**
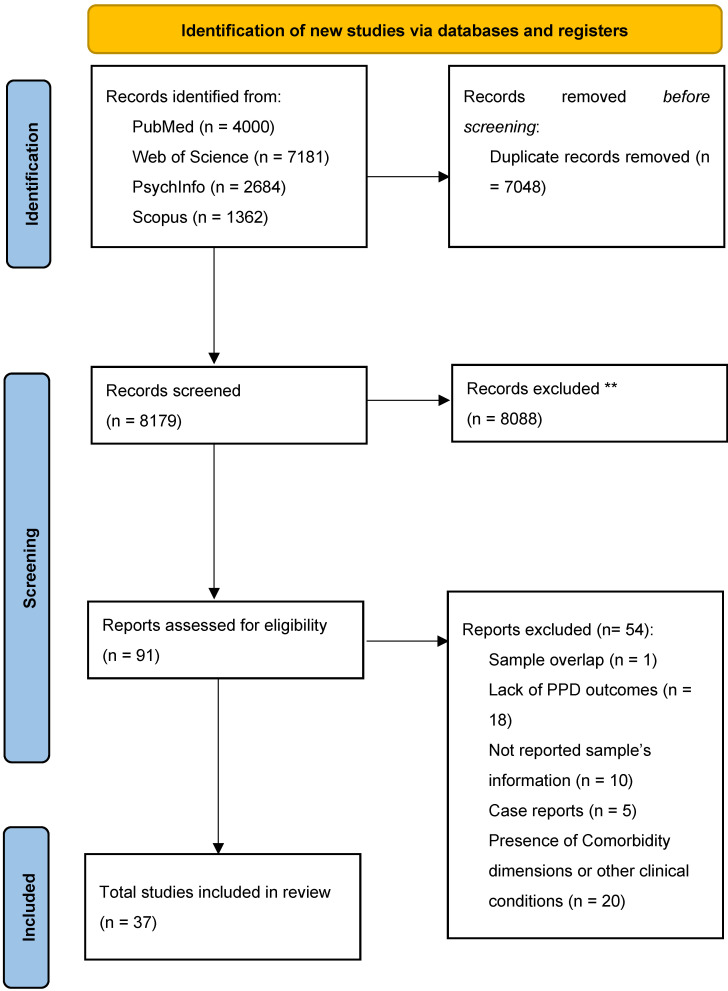
Flow chart showing the systematic process of revision. Reports excluded box in the flow chart may be reported overlap in exclusion criteria. ** indication provided by Rayyan support system.

Table 1 shows scripts and records retrieved from each database adopted for this systematic work. Figure 1 shows the process of revision.

Two independent reviewers screened, in a blind strategy with Rayyan software 2025, the overall records for title and abstract. Conflicts in the screening phase were solved via the search for agreement. For the phase of screening full-texts disagreements were solved by an independent reviewer (R.T.). The grade of agreement is reported in r = 0.91.

### 2.2. Eligibility Criteria

Studies were considered eligible if they met the following inclusion criteria: (i) empirical, observational, or longitudinal studies focused on analyzing the association between PPD and the clinical history of mothers of newborns; (ii) studies that included analysis, assessment or collection of information about the following psychiatric disorders in the history: major depressive disorder, obsessive–compulsive disorder, bipolar disorder or psychosis over a lifetime; (iii) studies identified or assessed the possible presence of a depressive disorder, postpartum depressive episode or postpartum depressive symptomatology. The following exclusion criteria were adopted: (i) studies concerning fathers; (ii) presence of health complications in pregnancy or postpartum, including fetal or infant complications; (iii) review studies, case reports, or study protocols unsuitable for risk analysis; (iv) studies that did not include an assessment of the PPD features through standardized tools (interviews or validated questionnaires); (v) absence of guidance on how the mother’s medical history was assessed (how the presence of psychopathological risk was assessed, at what stage of life, etc.). No limits of years of publication of the study were included in the record search.

### 2.3. Qualitative Assessment of Risk of Bias

**Figure 2:**
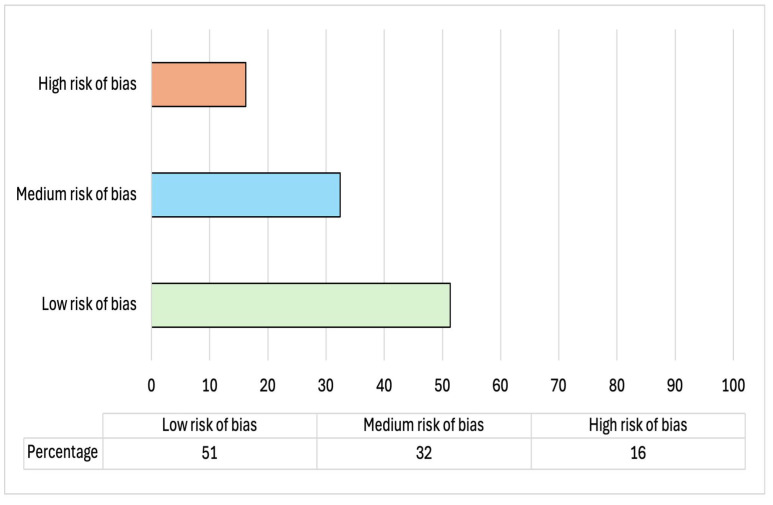
Percentage of the studies according to risk assessment.

**Figure 3:**
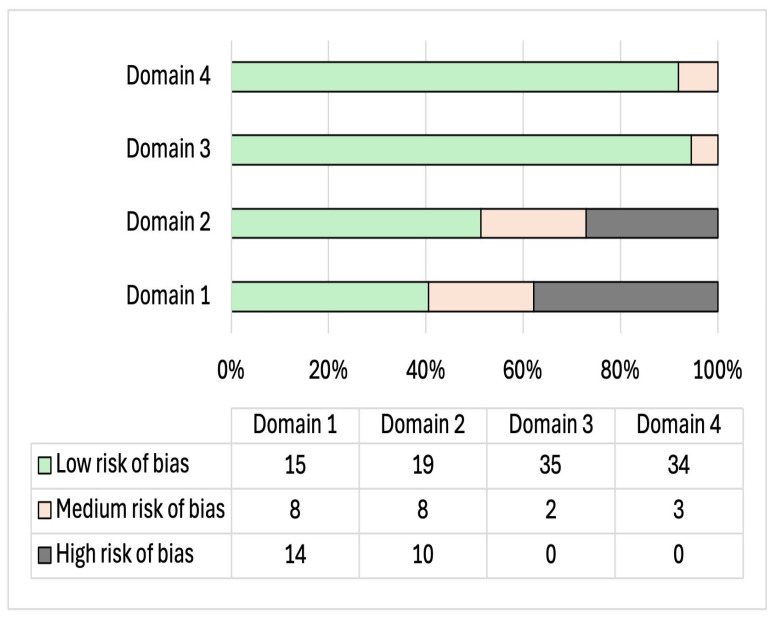
Risk of bias for each domain.

Each article included in the systematic review was assessed considering the quality of some specific dimensions aimed at reducing the risk of bias—as the risk of not sufficiently emphasizing the relevant results in relation to the objectives of the study—and ensuring monitoring of the systematic rigor of the work. This assessment was conducted through an adaptation of the Higgins’ tool for risk of bias (Higgins et al., 2019), structured to assess bias for the following domains: (I) sampling bias: the presence of small, unbalanced, or incomplete samples of data, including biases determined by assessments on critical samples, samples that are not representative of the general population (focused on specific ethnic groups, cultures or populations with social or psychological peculiarities), compromising the generalization; or the lack of demographic information that does not allow a clear generalization of the results; (II) method bias: due to the use of non-standardized or validated instruments, rather ad hoc created tools, questionnaire or poorly described interviews, not adequately guaranteeing reliability of tool and results; (III) results bias: due to unclear results, data not clearly explained in the results section; mainly adoption of qualitative analyses or lack of disclosure of the effect size; (IV) discussion bias: related to poor discussion or specification of the topic of interest, particularly referred to the aim of this systematic work. The analysis of each domain followed the following coding: no or low risk = 0; medium risk = 1; high risk = 2; according to two independent reviewers (F.F., S.T.), adopted to reduce the grade of the subjectivity of the evaluation. The risk of bias final evaluation for each study was calculated according to Table 2. The percentage of the study with low–medium–high risk was reported in a graphical summary. The quality assessment for each domain, useful to furnish us with information about the main strengths and weaknesses of the studies on this topic as well as a global indication of the characteristics of the studies included in this systematic review, was included in a graphical summary (Figure 2 in the Results Section), reporting the percentage of low-medium-high risk score for each domain (Figure 3 in the Results Section).

## 3. Results

### 3.1. Study Selection

From the 13,865 records retrieved from the databases, the elimination of duplicates yielded a total of 8179 records on which an initial selection by title and abstract was made. From this first stage, 91 articles were selected for full-text evaluation. Following the analysis of the inclusion and exclusion criteria and the qualitative assessment of the studies, 37 articles were finally eligible for inclusion in the systematic review (see Figure 1). Table 3 shows all characteristics relevant to the systematic review of the papers.

### 3.2. Risk of Bias

Qualitative analysis of the risk of bias of each study for the domains of (i) sampling, (ii) method, (iii) results, and (iv) discussion showed that 51 percent of the studies report a low risk of bias and 32 percent a medium risk of bias, underlining an overall good quality of the included studies (see Figure 2). It should be noted that 16 percent of the studies (n = 6) reported a high risk of bias. The analysis of the domains (see Figure 3) shows that this risk is attributable to the overall low quality of the studies in the *sampling domain* (only 40 percent of the studies, n = 15, report an absence of bias in this domain). Also, domain 2 (*method bias*) reported some criticisms in 27 percent of the study (10/37) due to the reported presence of ad hoc or not standardized questionnaires to assess the main variables of the study (Supplement 1 shows the specific assessment for each study).

### 3.3. Characteristics of the Studies

Studies included in this systematic review were carried out from 2002 (Freeman et al., 2002) to 2023 (Yu et al., 2023). The total sample size of the studies included approximately 1,064,089 mothers from 17 years of age (Guintivano et al., 2018; Long et al., 2020) to 49 years of age (Ongeri et al., 2018). The studies could present both a retrospective assessment (Ahmed et al., 2021; Alhasanat et al., 2017; Alsayed et al., 2021; Alzahrani, 2019; Balestrieri et al., 2012; Chojenta et al., 2016; Freeman et al., 2002; Mohammad et al., 2011; Peng et al., 2021; Silverman et al., 2020; Wu et al., 2022) of the mothers’ clinical condition, reported through anamnestic interviews or medical records that allowed the assessment of the association between PPD and previous diagnoses of mood disorders; either a longitudinal design (see Table 2), taking measurements in both the gestational and postnatal phase to assess trends in depressive symptoms and to check for correlations between depression during pregnancy and postnatal depression (e.g., Ahmed et al., 2021; Guintivano et al., 2018; Silverman et al., 2017, 2020).

The most widely used instrument for the assessment of PPD symptomatology was the Edinburg Postnatal Depression Scale (EPDS; Cox et al., 1987). According to the studies, the condition of postnatal depression was mainly assessed in relation to a history of depression during pregnancy or the presence of a major depressive disorder in the clinical history. Less explored was the association between PPD and the other disorders included in the systematic research (bipolar disorder, OCD, psychosis).

Of the studies that evaluated the possible presence of depression in pregnancy three of them reported information on the diagnosis (Underwood et al., 2017; Giardinelli et al., 2012; Mohamad Yusuff et al., 2015), nine studies measured depressive symptomatology without reporting a clinical diagnosis (Batmaz et al., 2015; De Venter et al., 2016; Kaźmierczak et al., 2020; Kubota et al., 2019; Lara et al., 2015; Tariq et al., 2021; Wu et al., 2022), five studies indicated that they controlled also for this variable without clarifying an eventual presence of clinical diagnosis (Choi et al., 2014; Dmitrovic et al., 2014; Leigh & Milgrom, 2008; Martínez-Borba et al., 2020; Ongeri et al., 2018), and five included women’s retrospectively self-reported diagnosis (Alhasanat et al., 2017; Chojenta et al., 2016; Peng et al., 2021; Tariq et al., 2021; Walker et al., 2021).

The history of clinical depression in the mother’s life was diagnosed either by accessing the women’s medical records (Guintivano et al., 2018; Silverman et al., 2017), via structured interview (SCID-I; Giardinelli et al., 2012) or by questionnaire such as the Inventory to Diagnose Depression (Zimmerman & Coryell, 1987) which examines the history of MDD based on the third version of the DSM (Kubota et al., 2019), the Mood Screener—Current/Lifetime self-report questionnaire, which follows the criteria of the DSM-IV (Carter et al., 2019) and the Major Depression Questionnaire (MDQ) that allow detecting current or past depression according to DSM-IV criteria (De Venter et al., 2016); in one case, how the diagnosis was found was not specified although the presence of it was reported (Ahmed et al., 2021); in 14 cases, it was self-reported by the participants (Underwood et al., 2017; Alhasanat et al., 2017; Alsayed et al., 2021; Alzahrani, 2019; Balestrieri et al., 2012; Batmaz et al., 2015; Kiviruusu et al., 2020; Lara et al., 2015; Leigh & Milgrom, 2008; Long et al., 2020; Milgrom et al., 2008; Mohammad et al., 2011; Peng et al., 2021; Tariq et al., 2021).

Few studies considered the bipolar disorder: in the study by Chan et al. (2021), hypomanic symptoms were considered therefore no diagnosis was made. In the studies by Freeman et al. (2002) and Perry et al. (2021a), on the other hand, the diagnosis of type I or type II bipolar disorder was used as a variable. Two studies also considered a diagnosis of obsessive–compulsive disorder (Giardinelli et al., 2012; Guintivano et al., 2018).

*The association between Ante-Natal Depression (AND) and PPD*

Twenty-three studies have examined the relationship between depression in pregnancy and postnatal depression. Of these studies, except Ongeri et al. (2018) who did not confirm a statistically significant association between the variables, all the authors confirmed a significant and positive correlation between depression in pregnancy and the risk of developing postpartum depression.

*The association between the History of Depression and PPD*

Twenty-one studies have examined the association between a history of clinical depression in mothers and the risk of developing postnatal depression. Fourteen studies confirmed a positive and statistically significant association between the two variables (see Table 2). Five studies did not find a significant association (Alhasanat et al., 2017; Carter et al., 2019; Chojenta et al., 2016; Kaźmierczak et al., 2020; Mohammad et al., 2011) and one study found a relationship between the variables only in those who also reported depression during pregnancy (Giardinelli et al., 2012). The presence of a clinical history of depression would seem to be associated with an increased risk of PPD. Only one study evaluates the presence of a previous PPD and a current PPD condition, confirming a significant correlation between the two variables (Alzahrani, 2019).

*Association between Bipolar Disorder, Psychosis, OCD and PPD*

From this systematic work emerges that the literature on the role of other psychiatric disorders as risk factors for PPD is poor. Among the few studies that could be included on the subject, Perry et al. (2021b) measured the possible correlation between types I and II of bipolar disorder and non-psychotic depression in the postpartum; however, they did not find significant results as there was no significant difference in the occurrence of non-psychotic postpartum depression episodes in women with and without psychotic symptoms during pregnancy. However, Chan et al. (2021) found a correlation between manic symptoms and depressive symptoms both during pregnancy and postpartum. In the study by Freeman et al. (Freeman et al., 2002), women with a clinical history of bipolar disorder reported more episodes of postpartum depression.

Guintivano et al. (2018) examined the correlation between OCD and postnatal depression and found a significant correlation; also, Giardinelli et al. (2012) found a significant correlation between perinatal depression and pre-pregnancy mental disorders, including 3.4% OCD. However, this association emerged only in those who presented higher depressive symptomatology during pregnancy.

*Beyond the psychopathology: other variables associated with PPD*

The analysis of the studies included in the review, in line with other previous work, beyond the reported risk associated with the psychological condition of the mothers, in terms of clinical history and antepartum conditions, revealed an important role of variables not directly related to the mother’s mental state. However, these clinical, social, demographic, or relational variables appear to influence the postnatal trajectory, modulating the relationship between previous and current psychological state. Among these, multiple studies have suggested a role of social class or socioeconomic status (Dmitrovic et al., 2014; Lara et al., 2015; Ongeri et al., 2018; Tariq et al., 2021; Wu et al., 2022), being unemployed or homemaker (Underwood et al., 2017; Balestrieri et al., 2012; Lara et al., 2015; Walker et al., 2021), lack of social and/or partner support (Alhasanat et al., 2017; Alzahrani, 2019; Giardinelli et al., 2012; Kaźmierczak et al., 2020; Lara et al., 2015; Milgrom et al., 2008; Mohammed et al., 2014; Mohammad et al., 2011), a family history of mental disorders (Peng et al., 2021; Silverman et al., 2020; Wu et al., 2022), having an unplanned pregnancy (Giardinelli et al., 2012; Lara et al., 2015; Mohammad et al., 2011; Tariq et al., 2021; Walker et al., 2021), advanced maternal age (Ahmed et al., 2021; Lara et al., 2015; Martínez-Borba et al., 2020; Silverman et al., 2017; Yu et al., 2023), breastfeeding problems (Chojenta et al., 2016; Van der Zee-van den Berg et al., 2021; Wu et al., 2022), medical variables concerning the mother (e.g., poor physical condition before pregnancy, being overweight) (Alzahrani, 2019; Mohammad et al., 2011; Van der Zee-van den Berg et al., 2021), medical variables concerning the child (Alzahrani, 2019; Walker et al., 2021; Wu et al., 2022), as well as poor sleep quality (Kiviruusu et al., 2020; Walker et al., 2021; Yu et al., 2023). All these aspects seem associated with a higher score of PPD symptomatology or a higher risk of developing a diagnosis of PPD.

According to the existing literature, one of the aspects that should be considered in the risk factors analysis of PPD is the life experiences of the mothers, which in the case of higher PPD symptomatology would be characterized by experienced trauma and/or sexual abuse (Guintivano et al., 2018; Walker et al., 2021) and having experienced stressful or adverse events over a lifetime (Underwood et al., 2017; Ahmed et al., 2021; Alhasanat et al., 2017; Alzahrani, 2019; Guintivano et al., 2018; Kiviruusu et al., 2020; Peng et al., 2021; Wu et al., 2022).

Other variables have been found to be significantly associated with PPD in some studies, although less explored generally across the literature, seem to be the following: having had a miscarriage in the previous pregnancy (Lara et al., 2015), cesarean birth (Lara et al., 2015), having a foreign nationality (Underwood et al., 2017; Balestrieri et al., 2012), being single (Lara et al., 2015), low educational status, (Dmitrovic et al., 2014; Lara et al., 2015), use or abuse of substances such as cannabis (Balestrieri et al., 2012), living in community centers (Balestrieri et al., 2012), having more than one child (Walker et al., 2021; Yu et al., 2023), having a family history of suicide (Lara et al., 2015), conflict with the partner or marital dissatisfaction (Kiviruusu et al., 2020; Lara et al., 2015; Ongeri et al., 2018), emotional distress during pregnancy and childbirth (Chojenta et al., 2016), affective ambivalence of the mother about their pregnancy journey (Martínez-Borba et al., 2020) parenting distress (Leigh & Milgrom, 2008), as well as low maternal self-efficacy (Van der Zee-van den Berg et al., 2021).

## 4. Discussion

Postpartum depression is a widespread condition that still needs clarity about the main risk factors involved in its onset, progress, and prognosis, as well as the prevalence rates, in general, and specific populations. Knowledge of psychiatric or psychopathological risk factors is generally important when dealing with a multidimensional and complex construct, and in the case of PPD, the past clinical picture could be a predictive factor of the severity of depressive symptoms and their prognosis. In this frame, and considering specific psychiatric risks, this systematic review clarified the following: 1. Depression in pregnancy is a risk factor for PPD; 2. It is more common for women who have had episodes of depression in their lifetime to be at higher risk for PPD; 3. In PPD diagnosis other variables, especially low socioeconomic status, having an unplanned/desired pregnancy, and the absence of social support, are more likely to contribute to an increase in the risk of association between clinical history and current condition. Surprisingly, few studies explored the impact of a family history of depression in influencing the current PPD, although it is well known the epigenetic impact of such a psychopathological condition (Luo et al., 2023). On the one hand, the environmental context affecting psychological state does not exclusively include social or relational dynamics, but it also includes clinical conditions of relatives that may negatively impact it. On the other hand, it is well known that mood disorders have a certain grade of heritability that should be considered from a psychodynamic perspective (Alameda et al., 2022).

Among psychological factors, several studies have indicated that women with a history of depression have a 6-fold higher risk of developing depressive symptoms in the postnatal period than women without a history of depression (de Castro et al., 2015). In light of the results of the following review, there is a general confirmation from empirical studies that a history of depression, depression in pregnancy, and postnatal depression are clinical dimensions that are associated. However, although this suggests that the psychiatric risk factor has some prognostic value, studies that did not report such inevitable association (Alhasanat et al., 2017; Carter et al., 2019; Chojenta et al., 2016; Kaźmierczak et al., 2020; Mohammed et al., 2014; Ongeri et al., 2018) might suggest that the psychopathological course is not inevitable and that intervening socio-relational variables would represent both protective or risk factors of a pathological trajectory influencing the incidence and diffusion in the specific condition of the disorder. Generally supportive family, friendship, and social context would appear to be a protective factor for the risk of PPD even in the presence of psychiatric risk factors. In this sense, PPD may be similar to other mood disorders reported in DSM, which are characterized, in terms of severity and prognostic outcomes by a complex expression of “epigenetic factors, i.e., a twist between both biological and personological predisposition factors that come into play and can be activated in unfavorable environmental conditions. These constellations may cause the cascade of negative events (i.e., symptoms) that represent, in the case of PPD, a healthy risk for the mother, the child, and the extended family context.

From this work emerges that episodes of mood disorders in a lifetime correlated significantly with the occurrence of PPD (Bolak Boratav et al., 2016; Byrne et al., 2014; Dekel et al., 2019), not only considering major depressive episodes, but also conditions such as bipolar disorder, obsessive–compulsive disorder, and psychosis, although few studies have investigated this risk (e.g., Chan et al., 2021; Freeman et al., 2002; Guintivano et al., 2018). Systematic, despite preliminary, confirmation of this evidence gives us the opportunity to emphasize how fundamental it is to carry out, during pregnancy, a good and all-inclusive assessment of the clinical history and an adequate psychiatric evaluation, with the aim of identifying possible markers of PPD risk and protecting both future mothers, children and the family system from the impact of pathology with potentially deleterious intra- and inter-individual implications (Guintivano et al., 2018).

The impact of PPD on the family system has been explored since the 1980s and several authors early have observed the interactions between mothers with depressive symptoms and their children (Tronick et al., 1978; Field et al., 1985; Beebe, 2005). Depression affects mothers’ behavior, limiting emotional expression and the quality of relational exchanges within mutually regulated dyadic interactions. The mother–child relationship varies along a continuum in which moments of high interactive synchrony alternate with moments of total lack of reciprocity (M. A. Bell, 2022). What is fundamental to the child’s development is the mother’s ability to dynamically regulate affectivity in this continuum, helping the children to build a core of positive affection and develop a representation of himself as effective. All these representations are fundamental to the development of a coherent sense of self and stable, secure relationships. In 1999, a meta-analysis by Beck confirmed the negative effects of maternal depression on the interactive mother–child relationship. Successively, other studies have shown that mothers suffering from PPD have great difficulty in correctly interpreting children’s signals to meet their primary physiological needs (Garset-Zamani et al., 2020) and that motherese, or baby speech, is also affected by maternal depression (Lam-Cassettari & Kohlhoff, 2020). Indeed, mothers with depressive symptoms often use flat language and tend to criticize the child, make hostile comments, or express annoyance at their play interactions (Ilyaz et al., 2024).

Overall, these characteristics of PPD directly affect child development (Marcos-Nájera et al., 2020; Dowse et al., 2020). Maternal depression has been found to be a significant risk factor for the development of an insecure attachment style, for the child’s social and emotional development, for the onset of internalizing and externalizing disorders in childhood, and for clinically significant outcomes in adolescence (Goodman & Tully, 2006; Silk et al., 2006). According to some authors, the predisposition to insecure attachment is linked to repeated experiences of failure in attempts at reciprocal affective regulation (Kordrostami & Kordrostami, 2019), probably due to the mother’s limited emotional availability to her child’s signals (Ierardi et al., 2019). Neurobiological studies have shown that such affective dysregulations in depressed mother–child interactions can lead to alterations in the child’s brain development, to a morpho-functional impairment of the right limbic system, due to the early loss of neural connections, predisposing the child to a greater vulnerability to the risk of developing psychopathologies later in life (Connell et al., 2015; Woody et al., 2016). In studies of older children, the consequences of PPD seem to involve not only the emotional dimension but also the behavioral and cognitive dimension, with problems in language development or the identification of special educational needs (Slomian et al., 2019). Various research, however, has emphasized that the negative effects on the child’s development depend mainly on the severity and persistence of the disorder (Slomian et al., 2019; Murray et al., 2001) and that developmental impairment is detectable when exposure to maternal depression persists for at least the first 6 months of life (Kordrostami & Kordrostami, 2019). More recently, maternal postnatal depression has also been examined in relation to its effects on the father and has been shown to be linked to a high risk of poor marital satisfaction, difficulty on the father’s part in dealing with his partner’s emotions (Cimino et al., 2022; Wang et al., 2021)and paternal depression (Thiel et al., 2022). Moreover, in a study conducted by Tian et al. (Tian et al., 2012), significant differences emerged between mothers with PPD and mothers without PPD. The first were significantly younger, had higher neuroticism scores, experienced more stressful life events such as sexual abuse in childhood, had fewer years of education, and had lower professional status. In these circumstances, PPD cases had a younger age of onset, more depressive episodes, and a longer maximum duration.

### Limitations

Despite the interesting evidence that emerged from this study some limits should be emphasized. First, almost 50% of the studies adopted, for assessing PPD, a self-reported questionnaire, such as the EPDS. This may generate substantial limits to the reliability and generalizability of the results, as reported also by the qualitative assessment of the review. In fact, EPDS assesses state mood condition, referring to the last seven days. In this sense, its score may be affected by extraordinary events occurring close to the measurement defining, in empirical research a sample bias if the depressive condition is not further explored. The EPDS should always be accompanied by a clinical interview, which was rarely reported by the authors of the studies examined.

Another limitation that could affect the reliability of the results of this systematic work is that the woman’s lifetime history of depression was self-reported in several studies. Therefore, the intensity and characteristics of the symptoms are unclear and the effect of time from past clinical events to the current state has not been controlled. Also, multiple variables were not controlled with respect to these aspects due to the self-reported nature of the data.

Another important limit that should be emphasized is that there is a clear lack of studies analyzing the relationship between other psychiatric disorders and PPD (see our evidence on obsessive–compulsive disorder, psychosis, and bipolar disorder) or other trait dimensions (Cimino et al., 2023), even though epidemiological data suggest an increase in cases of these disorders in the general population, which may therefore also affect the course of pregnancy and post-pregnancy.

Finally, a further limitation is the absence of follow-ups. There is a lack of evidence that would allow us to state unequivocally that the presence of psychiatric disorders, or episodes of psychiatric disorders, prior to pregnancy may be a risk factor for symptom exacerbation and an inauspicious prognostic course in relation to PPD over the first months of motherhood. These limitations are physiologically due to the characteristics and heterogeneity of the studies that as the main limit of this work did not allow us to quantitatively explore the effect size of the evidence via a meta-analysis. The exclusively qualitative check of the studies included in the review furnished us with the domain that should be improved in the protocol study on this topic, but it was characterized by a grade of subjectivity and lack of quantitative data that did not allow us to furnish strong and generalized evidence.

## 5. Conclusions

Peripartum depression also has considerable effects on the social level. In fact, the Italian Superior Institute of Health (ISS; Camoni et al., 2023) reported that in 2023 the cost of peripartum depression was approximately €88,000 per woman. The largest costs are due to the negative impacts of maternal depression on the child and refer to health care, developmental difficulties, and mental and educational disorders. On the other hand, mother-related costs are due to a lack of productivity and poor quality of life due to health conditions (Bauer et al., 2016). Accordingly, taking into consideration the consequences of PPD on the mother, child, parental couple, and extended society, it would be crucial to structure screening programs in pregnancy, to identify the most vulnerable individuals and structure an intervention to reduce the risk associated with the presented variables. This would bring numerous benefits both at an individual level, for the mother and the child’s well-being, and at a social level, reducing the cost of depression’s impact on the health of both mothers and children (Tambelli et al., 2022).

It is certainly important for the clinician to promote the mother’s psychological well-being starting from the social support during the first and most uncertain stages of caring for a newborn (Ierardi et al., 2019). Also, work on the psychological skills of the mothers would be relevant. Monti and Agostini (Vismara et al., 2016) highlighted some characteristics that can have a positive influence on the process of adapting to motherhood: good maternal self-esteem and coping skills, as well as the child’s considered ‘easy’ temperament. As suggested by this systematic work, while it is important to focus on protective factors, it is also important that these are included in a comprehensive assessment of mental health status to highlight a possible role of psychiatric risk factors. This would allow the development of a personalized approach, considering the vulnerabilities and resources of each mother.

## Figures and Tables

**Table 1:** Scripts and records for each explored database.

Database	SCRIPT	N
PubMed	postpartum depression OR perinatal depression OR post-natal depression OR PPD OR PND AND bipolar disorder OR depression OR major depressive disorder OR schizophrenia OR psychotic symptom * OR obsessive compulsive disorder OR DOC	4000
Web of Science	postpartum depression OR perinatal depression OR post-natal depression OR PPD OR PND AND bipolar disorder OR depression OR major depressive disorder OR schizophrenia OR psychotic symptom * OR obsessive compulsive disorder OR DOC	7181
Psychinfo	postpartum depression OR perinatal depression OR post-natal depression OR PPD OR PND AND bipolar disorder OR depression OR major depressive disorder OR schizophrenia OR psychotic symptom * OR obsessive compulsive disorder OR DOC	2684
Scopus	postpartum depression OR perinatal depression OR post-natal depression OR PPD OR PND AND bipolar disorder OR depression OR major depressive disorder OR schizophrenia OR psychotic symptom * OR obsessive compulsive disorder OR DOC	1362
TOTAL RECORDS		13,865

**Table 2:** Conditions for the quality assessment of each study.

	**Conditions**
Low Risk of Bias	1. All the domains = 0; 2. Half of the domains = 1.
Medium Risk of Bias	1. Up to half of the domains = 1; 2. Only one domain = 2 and the other domains = 0 or one domain = 1.
High Risk of Bias	1. Up to one domain = 2; 2. Only one domain = 2 and more the one of other domains = 1.

**Table 3:** Main characteristics of the studies included in this systematic review.

	Authors (Year)	Country	Sample Size	Age (st.dev)/ Age Range	Time Assessment	Risk Factors	PPD Diagnosis (Y/N)	Assessment Tool for PPD	Other Variables Considered or Controlled	Main Results
1	Ahmed et al. (2021)	Egypt	257	27.98 (sd = 4.7)	Between 2 and 6 months after birth	Clinical History of Depression (Non-specified)	No	EPDS	Sociodemographic variables: socioeconomicual status, pregnancy conditions, breastfeeding information.	PPD is associated with depression in clinical history if considered in association with other risk variables (from demographic data).
2	Alhasanat et al. (2017)	USA (Arabian Women)	50	30.16 (sd = 6.45)	Between 1 and 6 months after birth	Clinical History of Depression (Self-reported) Antenatal Depression (and Anxiety) (Self-reported)	Not specified	EPDS PDPI-R (*Postpartum Depression Predictors Inventory*)	Sociodemographic variables, Social Support, Pregnancy condition, Life stress conditions, Child’s Temperament	Antenatal condition of depression (and anxiety) is associated with PPD.
3	Alsayed et al. (2021)	Saudi Arabia	172	29 (sd = 5)	Between 1 and 4 months after birth	Clinical History of Depression (Self-reported)	Y	EPDS	Sociodemographic variables, Social Support, Pregnancy condition, Life stress conditions, Family History	Clinical history of depression and other risk factors may predict PPD.
4	Alzahrani (2019)	Saudi Arabia	217	33.2 (sd = 4.5)	Not specified	Clinical History of Depression (Self-reported)	Y	EPDS	Sociodemographic variables, Social Support, Problems during pregnancy and childbirth.	Clinical history of postpartum depression is associated with PPD.
5	Balestrieri et al. (2012)	Italy	1608	32.2 (sd = 4.8)	Between 12 and 15 weeks after birth	Clinical History of Depression (Self-reported)	Y	EPDS	Sociodemographic variables, Clinical variables.	Clinical history of depression is associated with higher scores in EPDS.
6	Batmaz et al. (2015)	Turchia	276	28.8 (sd = 5.6)	T0: during pregnancy; T1: 24 weeks after birth.	Clinical History of Depression (Self-reported) Antenatal Depression (Assessed with BDI).	Y	EPDS	Sociodemographic variables, Work status, Pregnancy information	BDI and EPDS scores are positively correlated. PPD is more severe in those who experienced a clinical history of depression.
7	Carter et al. (2019)	USA	1796	28.32 (sd = 5.51)	Antenatal assessment: T1:1° trimester; T2: 2° trimester; T3: 3° trimester. Postpartum: T1: 12 weeks; T5: 27 weeks; T6: 40 weeks.	Clinical History of Depression (Mood Screener—Current/Lifetime)	Y	EPDS Mood Screener—Current/ Lifetime.	Sociodemographic variables, Pregnancy information	Antenatal depression (AND) is associated with higher scores at the EPDS in post-natal assessment. However, in longitudinal assessment for AND there is a faster decreasing of EPDS score.
8	Chan et al. (2021)	Hong Kong	N T1: 229 N T2: 97 N T3: 56	18–30, 31–35 e ≥36.	T1:1° trimester; T2: 2° trimester; T3: 6 weeks after birth.	Depressive Symptoms Hypomaniacal Symptoms	N	Self-report: Hypomania Checklist Hospital Anxiety and Depression Scale.	Sociodemographic variables, Educational Levels	Hypomaniacal symptoms are related to antenatal and postnatal depression.
9	Choi et al. (2014)	Sud Korea	467	32	T1:1° trimester; T2: 2 months after birth.	Antenatal Depression (EPDS)	Not specified	EPDS	Sociodemographic variables, Pregnancy information	AND is associated with risk for PPD.
10	Chojenta et al. (2016)	Australia	5219	Birth years: 1973–1978	5 survey in the years 1996, 2000, 2003, 2006, 2009	Clinical History of Depression (Self-reported) Antenatal Depression (Self-reported)	Y (self-reported)	Self-reported Diagnosis of EPDS	Sociodemographic variables, Physical condition, Pregnancy information	AND predicts PPD. clinical history of antenatal depression does not predict PPD.
11	De Venter et al. (2016)	Belgio	183	30	T0: 3° trimester of pregnancy; T1: 12 weeks after birth; T3: 24 weeks after birth.	Clinical History of Depression (MDQ) AND (EPDS)	Y	EPDS Major Depression Questionnaire (MDQ). Depression Anxiety and Stress Scales (DASS-D).	Childhood traumatic experiences	AND episodes are predictors of PPD.
12	Dmitrovic et al. (2014)	Serbia	195	Not specified	T0: 3° trimester of pregnancy; T1: 6–8 weeks after birth.	AND (EPDS)	Not specified	EPDS	Sociodemographic variables Pregnancy information	Strong correlation between AND and PPD.
13	Freeman et al. (2002)	USA	50	43.3 (sd:11.4)	Not specified	Bipolar Disorder (Type I and Type II)	Y	Clinical Interview (DSM-IV)	Womens’ hormones	Episodes of bipolar disorder are associated with more PPD.
14	Giardinelli et al. (2012)	Italy	590	34 (sd = 4.2)	T0: 28–32 of pregnancy; T2: 12 weeks after birth.	Mood and Anxiety Disorders (SCID-I) AND (SCID-I, EPDS)	Y	EPDS	Sociodemographic variables Pregnancy information	Correlations between mood and anxiety disorders (DOC) before pregnancy, EPDS score during pregnancy, and EPDS scores postpartum only in women with AND diagnosis.
15	Guintivano et al. (2018)	USA	549	17–45 anni (26.7)	6 weeks after birth.	Psychiatric History (Medical Archives)	Y	EPDS	Traumatic Life experiences	History of clinical depression is one of the main predictors of PPD. Also, a DOC lifetime diagnosis seems to be related to PPD
16	Kiviruusu et al. (2020)	Poland	1670	30.7 (sd: 4.6)	T0: 32° week of pregnancy; T1: 3 months after birth; T2: 8 months after birth; T3: 24 months after birth.	Depressive symptoms (Self-reported)	Y	CES-D	Sociodemographic variables, Lifestyles habits Social support	History of depression predicts PPD.
17	Kaźmierczak et al. (2020)	Poland	106	29.15 (sd = 4.79)	T0: 37° week of pregnancy; T1: 6 weeks after birth.	Clinical History of Depression (Self-reported) Antenatal Depressive Symptoms (EPDS)	N	EPDS	Sociodemographic variables	AND is associated with PPD. Clinical history does not seem to be associated with current PPD.
18	Kubota et al. (2019)	Japan	338	38.6 (sd = 4.7)	T0: during pregnancy (average weeks: 22.7 (sd:6.3)); T1: 1 month after birth.	Clinical History of Depression (IDDL) Antenatal Depressive Symptoms (EPDS)	Y	EPDS	-	Clinical history of major depressive disorders experienced higher AND and PPD. AND affects PPD.
19	Lara et al. (2015)	Mexico	210	29.50 (sd = 6.34)	T0: 26° week of pregnancy; T1: 6 weeks after birth; T2: 6 months after birth.	Depressive Symptoms (PHQ-9)	Y	SCID PHQ-9	Sociodemographic variables, Pregnancy information, Social support, Traumatic life Experience	Antenatal Depressive symptoms are associated with PPD. PPD is associated with other risk variables (social support, traumatic experiences)
20	Leigh & Milgrom (2008)	Australia	161	30.8 (sd: 5.1)	T0: 26–34 of pregnancy; T1: 10–12 weeks after birth.	Clinical History of Depression (Self-reported: Demographics and Psychosocial Risk Factors Questionnaire) Antenatal depression (EPDS, BDI)	Not specified	BDI	Sociodemographic variables, Social support, Parental stress index, Cognitive style self-esteem	In a complex predictive model including all the variables, clinical history of depression and parental stress are significant predictors of PPD.
21	Long et al. (2020)	USA	557	17–44	T0: first midwife appointment; T1: 6 weeks after birth.	Clinical History of Mental Health (Self-reported) Antenatal Depressive symptoms (EPDS)	Y	EPDS	Sociodemographic variables,	Clinical history of depression is associated with PPD. AND is associated with a higher score of EPDS after birth.
22	Martínez-Borba et al. (2020)	Spain	101	32.59 (sd = 4.39)	T0: 16–36 weeks of pregnancy; T2: 2–4 weeks after birth;	Antenatal Depression (BDI-II)	Not specified	Beck Depression Inventory II	Bio–psycho–social factors	AND and PPD significantly correlated.
23	Milgrom et al. (2008)	Australia	22,968	30.3 (sd = 5.6)	T0: during pregnancy; T2: 6° weeks after birth.	Clinical History of Depression (Self-reported) Antenatal Depression (EPDS)	Y	EPDS	Sociodemographic variables, Antenatal medical condition, Childhood abuse	PPD is associated with clinical history of depression and depressive symptoms, and AND.
24	Mohammed et al. (2014)	Egypt	200	29 (sd = 5.2)	Between 1 and 14 months after birth	Clinical History of Depression (Self-reported)	Y	EPDS	Sociodemographic variables, Social support, Perinatal conditions	Clinical history of depression is not related to PPD.
25	Mohammad et al. (2011)	Giordania	353	18–45	T0: 3° trimester of pregnancy; T1: 6–8 weeks after birth; T2: 6 months after birth.	Antenatal Depression (EPDS)	N	EPDS DASS-21 (*Depression Anxiety and Stress Scale*)	Sociodemographic variables, Social support, Stress levels, Economic problems	AND is associated with PPD.
26	Mohamad Yusuff et al. (2015)	Malaysia	2072	26.7 (sd = 5.6)	T0: 36–38 weeks of pregnancy; T1: 1 month after birth; T2: 3 months after birth; T3: 6 months after birth.	Antenatal Depression (EPDS)	Y	EPDS	Sociodemographic variables, Social support, Maternal worrying, Partner’s support	AND is associated with PPD. The role of the partner affects the relationship.
27	Ongeri et al. (2018)	Kenia	171	18–49	T0: 3° trimester of pregnancy; T1: 6–10 weeks after pregnancy.	Antenatal Depression (EPDS)	Not specified	EPDS	Sociodemographic variables, Pregnancy information, Social support, Economic problems, Partner’s support	AND is associated with PPD but the association is not statistically significant.
28	Peng et al. (2021)	China	4813	29	6 months after birth	Antenatal Depression (Self-reported)	Y	EPDS	Sociodemographic variables, Pregnancy information, Family history of mental conditions, Stressful events in life, Breastfeeding	AND is associated with an increased risk of PPD. Significant role of life events and familiarity with mental health problems.
29	Perry et al. (2021b)	UK	124	>18 years of age	T0: 12° week of pregnancy; T2: 24 sett gravidanza; T3: 3 mesi postpartum.	Antenatal Condition (SCAN): Bipolar Disorder; Not Psychotic Depression; Hypomania.	Y	SCAN Interview	Sociodemographic variables, Previous pregnancy and childbirth	No association between bipolar disorder and PPD. PPD with no psychotic symptoms is not associated with other psychotic conditions reported in antenatal assessment.
30	Silverman et al. (2017)	Sweden	707,701 (Data collection between 1997 and 2008)	15–41	In the first years of the child	Clinical History of Depression (Medical Archives)	Y	Swedish National Patient Register	Perinatal conditions	Clinical history of depression increases the risk for PPD.
31	Silverman et al. (2020)	Sweden	707,701 (Data collection between 1997 and 2008)	15–41	In the first years of the child	Clinical History of Mental Health with or Without Psychotic Events (Medical Archives)	Y	Swedish National Patient Register		PPD rate is higher in women with history of depression, especially in case of psychotic conditions.
32	Tariq et al. (2021)	Pakistan	200	27.1 (sd: 5.08)	T0: 1° trimester of pregnancy; T1: 2 weeks after birth.	Antenatal Depression (EPDS)	N	EPDS	Sociodemographic variables, Previous pregnancy experiences	AND is a significant predictor of PPD. There is a role of sociodemographic variables.
33	Underwood et al. (2017)	New Zealand	5301	30.2 (sd = 5.8)	T0: 3° trimester of pregnancy; T1: 9 months after birth.	Clinical History of Depression (Self-Reported) Antenatal Depression (EPDS)	Y	EPDS	Sociodemographic variables, Lifestyle habits	AND increase the risk of PPD diagnosis.
34	Van der Zee-van den Berg et al. (2021)	Netherlands	1406	30.6	T0: 3 weeks after birth; T2: 1 months after birth; T3: 12 mesi post dati su eventi di vita.	Clinical History of Depression (Self-Reported) Antenatal Depression (EPDS)	Not specified	EPDS	Sociodemographic variables, Lifestyle habits, Medical conditions, Pregnancy information	Clinical history of depression is associated independently with PPD. AND is a risk for PPD
35	Walker et al. (2021)	Netherlands	5109	31.5 (sd = 4.1)	T0: 16° weeks of pregnancy; T1: 13 weeks after birth.	Antenatal Depression (CES-D)	Not specified	CES-D	Sociodemographic variables, Pregnancy condition	AND is associated with PPD.
36	Wu et al. (2022)	China	296,873	29 (sd = 4.7)	From 14 days to 42 days after birth	Antenatal Depression (Self-reported)	Y	EPDS	Sociodemographic variables, Pregnancy condition, Stressful life events, Child’s health status	AND is associated with PPD
37	Yu et al. (2023)	China	578	30.24 (sd = 3.331)	T0: 28–34 weeks of pregnancy; T2: 6 months after birth.	Antenatal Depression (EPDS)	Not specified	EPDS	Sociodemographic variables, Lifestyles habits, Previous pregnancy, experiences	AND is associated with risk of PPD

## Supplementary Materials

The following supporting information can be downloaded at: https://www.mdpi.com/article/10.3390/bs15020173/s1, Supplement 1, Dataset of qualitative assessment.
